# Plant pest surveillance: from satellites to molecules

**DOI:** 10.1042/ETLS20200300

**Published:** 2021-03-15

**Authors:** Gonçalo Silva, Jenny Tomlinson, Nawaporn Onkokesung, Sarah Sommer, Latifa Mrisho, James Legg, Ian P. Adams, Yaiza Gutierrez-Vazquez, Thomas P. Howard, Alex Laverick, Oindrila Hossain, Qingshan Wei, Kaitlin M. Gold, Neil Boonham

**Affiliations:** 1 Natural Resources Institute, University of Greenwich, Central Avenue, Chatham Maritime, Kent ME4 4TB, U.K.; 2 Fera Science Ltd., York Biotech Campus, Sand Hutton, York YO41 1LZ, U.K.; 3 School of Natural and Environmental Sciences, Agriculture Building, Newcastle University, King's Road, Newcastle upon Tyne NE1 7RU, U.K.; 4 International Institute of Tropical Agriculture, Dar el Salaam, Tanzania; 5 Department of Chemical and Biomolecular Engineering, North Carolina State University, Raleigh, NC 27695, U.S.A; 6 Plant Pathology and Plant Microbe Biology Section, Cornell University, 15 Castle Creek Drive, Geneva, NY 14456, U.S.A

**Keywords:** biosecurity, diagnostics, phytopathology, surveillance

## Abstract

Plant pests and diseases impact both food security and natural ecosystems, and the impact has been accelerated in recent years due to several confounding factors. The globalisation of trade has moved pests out of natural ranges, creating damaging epidemics in new regions. Climate change has extended the range of pests and the pathogens they vector. Resistance to agrochemicals has made pathogens, pests, and weeds more difficult to control. Early detection is critical to achieve effective control, both from a biosecurity as well as an endemic pest perspective. Molecular diagnostics has revolutionised our ability to identify pests and diseases over the past two decades, but more recent technological innovations are enabling us to achieve better pest surveillance. In this review, we will explore the different technologies that are enabling this advancing capability and discuss the drivers that will shape its future deployment.

## Introduction

With United Nations figures estimating that the world population is expected to reach almost 10 billion people in 2050 [1], there will be growing demand for food delivered through increased production, changes in dietary preferences and improved distribution [2]. Plant pests and diseases pose a significant constraint to agricultural productivity, with estimated losses of 1/3 of global yield [3,4].

Climate change, global trade (including internet sales) of plants and plant parts, movement of people, and changes to agricultural systems are major drivers for plant disease emergence [5–9]. New and emerging diseases are frequently highly significant as management measures are often not in place, resulting in devastating consequences for food security and rural economies. For example, the fall armyworm (FAW) (*Spodoptera frugiperda*), native to tropical and subtropical regions of the Americas, was recently introduced to sub-Saharan Africa where it has been estimated to cause yield losses in maize of 21–53%, worth 2.5–6.1 billion USD annually across 12 countries where maize is the most widely grown staple food crop [10]. *Xylella fastidiosa*, a bacterial plant pathogen prevalent across the United States, was first reported in Europe in 2013 in olive trees in Italy [11]. It is transmitted by xylem feeding insect vectors and causes diseases in a wide range of hosts including crops such as grapevine and citrus, trees and ornamental plants [12,13]. *X. fastidiosa* is a significant threat to agricultural ecosystems, resulting in dramatic changes to landscapes and cultures [9]. Endemic pests can be as damaging as emerging diseases. For example, late blight disease, caused by *Phytophthora infestans*, is problematic wherever potato is grown, and losses are estimated at 15% of the global yield, worth approximately 5 billion Euro annually [14].

Developments in technology, particularly methods based on molecular biology, have improved our ability to detect and identify pests and pathogens [15] and diagnose the causal agents of disease [16–18]. Technologies more suited to surveillance and monitoring, on the other hand, have only more recently seen significant advances. In a biosecurity context, effective surveillance strategies enable early detection in new regions, management of eradication strategies and mapping of disease spread, and provide data on freedom from a pest in support of exclusion zones and containment strategies. For endemic pests, effective monitoring facilitates the timely deployment of management measures.

In this review, we provide an overview of the currently available technologies that are revolutionizing in-field plant disease detection and discuss how these technologies can be used individually or in combination to support biosecurity and pest surveillance. The technical challenges of each technology have been extensively reviewed previously (for example, [8,19,20]); here we will address deployment of technologies for different detection scenarios and discuss future research and development directions that could accelerate adoption and assist surveillance and the prediction and prevention of future plant disease outbreaks.

## Remote sensing

Plant disease changes how solar radiation interacts with leaves, canopy, and plant energy balance, which can be detected with proximal (non-aerial) and remote (aerial) sensing [21,22]. Remote sensing is the use of non-ground-based imaging systems to obtain information about processes and events on Earth, and is unique among the detection and diagnostic methods discussed herein in its ability to offer passive monitoring for the disease at scale, rather than active sampling. Most fields are too large for growers to cost-effectively monitor frequently; remote sensing enables high temporal- and spatial-resolution monitoring that can be used to deploy high-accuracy ground diagnostics and remediation activities to diseased plants before epidemics emerge.

Broadband and multispectral methods relying primarily on visible (VIS) and near-infrared (NIR) reflectance indices, such as normalised difference vegetation index (NDVI), have been used to sense late-stage plant disease since the 1980s [23–25]. Despite this long-established proof of concept, plant disease remote sensing has remained underdeveloped in comparison with other types of agricultural sensing, such as water usage and monitoring nutrient deficiency. This is because historically accessible datasets have lacked the spatial, temporal, and/or spectral resolution to facilitate early disease intervention. For example, Landsat-8 and Sentinel-2 imagery can indirectly identify variation suggestive of disease but are non-diagnostic and non-actionable for most growers due to their coarse spectral and spatial resolutions. Recent advances in remote sensing technologies, methodologies, and analytics have helped to resolve some of these historical challenges. Commercial satellites, such as Planet Labs and Maxar, for example, now enable frequent, precise returns with high spatial and temporal resolution with growing spectral resolution and have been recently established to be capable of disease monitoring [26,27]. Unmanned aerial vehicles (UAV, or ‘drones') and low-altitude aircraft are well established to be capable of disease detection and monitoring, though reliable differentiation and diagnosis remains challenging [28–32]. Access to these sorts of imagery is now widely available to growers due to the growing number of service providers offering weekly to sub-weekly imagery satellite, aerial and/or UAV imagery. Some example providers include Climate Corporation, VineView, Taranis, and Ceres Imaging, with a multitude of smaller providers offering UAV imagery to niche markets.

The advent of more widely available multi- and narrowband visible to shortwave infrared (SWIR; 400–2500 nm) proximal and remote sensing has reinvigorated the discipline of plant disease sensing with its newly established capacity for pre-symptomatic disease detection [32–37] and differentiation [38,39].

## Detection of the disease using smartphone image analysis

The combination of imaging, telecommunications and computing technology in modern smartphones has opened a multiplicity of opportunities for their use as diagnostic tools that can capture, display, and share results almost instantly. Most types of crop pests and diseases cause visible symptoms that can be recognized by a trained person, but few such people are accessible to farmers, particularly in the developing world. To overcome this, a smartphone can be used to capture an image of the symptoms which is then sent via the internet to a platform where human experts can make the diagnosis [40–42]. An alternative strategy is based on artificial intelligence and machine learning methods that enable direct detection and identification of pests on-site.

**Figure 1.**
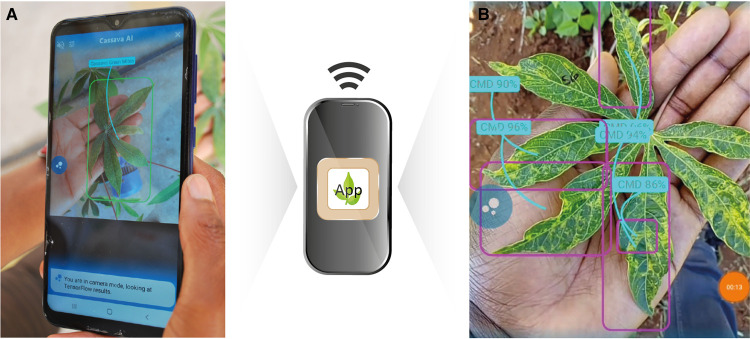
Smart phone applications facilitate wider access to diagnostic tools. Example of the application of the PlantVillage Nuru App for identification of (**A**) cassava green mites and (**B**) Cassava mosaic disease (CMD) in cassava leaves.

Several free smartphone apps have been developed using these technologies for a wide range of crop/pest combinations. Leaf Doctor [43] is a system for performing quantitative assessments of plant diseases; Pestoz (Creotix, India) diagnoses diseases from images of vegetable and crop plants with a primary focus on India; Plantix (PEAT GmbH, Germany) is a system for diagnosis of diseases, pest damage and nutrient deficiencies on crops; PlantVillage Nuru [44,45] (Figure 1) is a system for diagnosing viral diseases of cassava and damage caused by FAWs on maize; and Tumaini (International Center for Tropical Agriculture—CIAT, Colombia) [46] is a system for identifying diseases affecting banana plants. These apps also provide a vehicle for delivering advice for the management of the identified pest. PlantVillage Nuru and Tumaini can be used without an internet connection, which is an invaluable feature when used in remote, rural locations.

Conditions such as the quality and quantity of images used to train the model and the variation and complexity of the disease symptoms can influence the accuracy (sensitivity and specificity) of the diagnostic system [47]. In-field conditions, such as sunlight, wind and rain, can also affect the accuracy of the diagnosis system. Mrisho *et al*. [47] and Ramcharan *et al*. [45] showed that the in-field accuracy of PlantVillage Nuru using one leaf (21–59%) was lower than the on-screen accuracy (43–81%). When the number of leaves inspected by PlantVillage Nuru was increased to six per plant, however, accuracy increased to 93% for cassava mosaic disease, 73% for cassava brown streak disease and 93% for cassava green mite [47].

Information on the accuracy (both in-field and on-screen) of Leaf Doctor, Pestoz, and Plantix has not been made public while the on-screen accuracy of Tumaini was reported to range between 70% and 99% [46].

There is limited information on the comparison of digital diagnostic apps to other diagnostic methods, however; the main alternative diagnostic method for plant diseases is through visual inspection of the plant either by farmers or agricultural extension officers. Using a standard set of 170 single cassava leaf images, Mrisho *et al*. [47] showed that PlantVillage Nuru could diagnose symptoms of diseases at an accuracy (64%) higher than that of agricultural extension officers (50%) and cassava farmers (42%), suggesting that diagnostic apps such as this could offer a means to improve the diagnostic capability of users. Nevertheless, compared with other testing approaches, large amounts of data can be collected without significantly adding to the cost of surveillance. As a result, these apps are already being used to map the occurrence and spread of pests and diseases, which is contributing to forecasting and pest risk assessment (unpublished data).

## Pathogen detection using field-portable VOCs sensors

Volatile organic compounds (VOCs) emitted from plants have recently emerged as noninvasive diagnostic markers for the detection of pathogens and pests [48–52]. Laboratory-based techniques such as gas chromatography-mass spectrometry (GC–MS) [53] have slow turnaround times which have limited uptake. Direct field measurement of plant biogenic VOCs allows a more accurate assessment of VOC fluxes emitted under natural conditions, and several approaches have been explored in recent years.

Miniature GC–MS adapts the same detection mechanism as laboratory instruments: examples include the ion mobility spectrometer (IMS) [54], the ion-trap MS (TMS) [55] and the differential mobility spectrometer (DMS). A miniature GC-DMS device was developed for rapid differentiation of VOCs from healthy and *Phytophthora ramorum*-infected rhododendron plants [56] and a fully automated light-weight GC device was demonstrated to distinguish healthy and aphid-infested milkweed (*Asclepias syriaca*) with >90% accuracy [57].

Electronic-noses (e-noses) are artificial olfaction devices that utilise an array of cross-reactive gas sensors to produce a fingerprint response to a given gaseous molecule or mixture without identifying individual compounds [58–60]. E-noses are appealing as in-field systems for the rapid detection of plant VOCs and have been used for the diagnosis of diseases including fire blight (*Erwinia amylovora*) and blossom blight (*Pseudomonas syringae* pv. *syringae*) in asymptomatic apple trees [61–63] and early blight disease/gray mold disease of tomato seedlings [64]. E-nose devices have several limitations restricting their adoption as a diagnostic tool including signal drift, susceptibility to humidity, moderate sensitivity, complex data processing, and high purchase cost.

**Figure 2.**
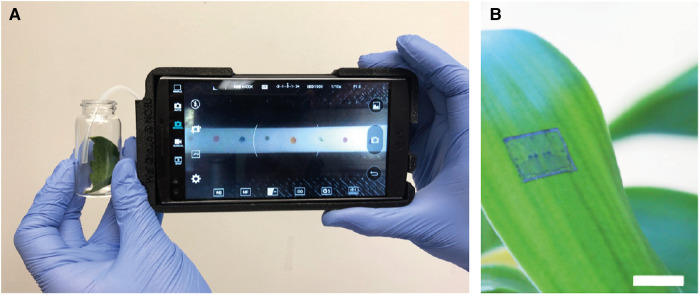
Miniaturised sensors enable in-field detection. (**A**) A smartphone-based VOC sensor for early detection of late blight (*P. infestans*) and differentiation of early blight and Septoria leaf spot on tomato [65]. (**B**) A carbon nanotube-graphite-based wearable sensor transferred onto the surface of a live leaf [70].

A smartphone-based gas sensor developed for noninvasive diagnosis of plant diseases has been demonstrated by Li *et al*. [65] (Figure 2A), combining brightfield smartphone microscopy with a paper-based colorimetric chemical sensor array [66,67] to achieve high sensitivity for green leaf volatiles and phytohormones. This device was able to identify late blight (*P. infestans*) in asymptomatic plants 2 days post inoculation and accurately differentiate late blight from other pathogens. The integration of ‘wearable’ sensors on leaves or stems of plants (Figure 2B), holds great promise for real-time and wireless monitoring of plant health [68,69], including continuous monitoring of plant VOCs [70].

## Protein diagnostic platform: lateral flow assay (LFA)

Lateral flow assays (LFAs) are simple, rapid, and cost-effective immunochromatographic tests (exemplified by home pregnancy tests) that allow the detection of proteins of interest without the requirement for equipment or technical expertise [71].

**Figure 3.**
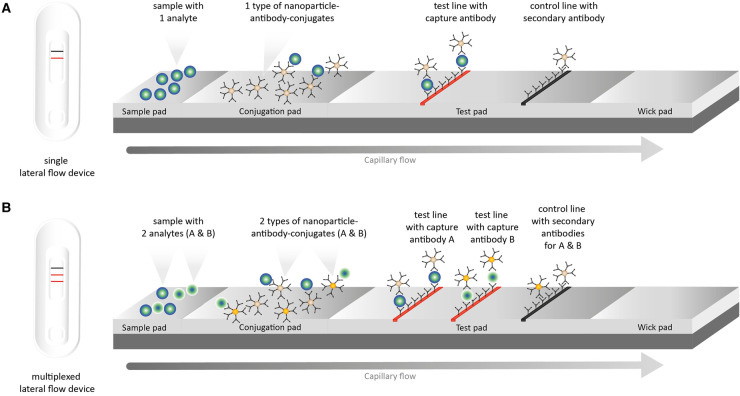
Multiplexing enables more data to be generated from each sample. Schematic representation of (**A**) single and (**B**) multiplexed lateral flow devices (LFDs). LFDs are strips in a plastic cassette. Each strip is composed of four membranes (i.e. sample, conjugation, test and wick/adsorbent pad) on a plastic adhesive backing card. The conjugation pad contains detection antibodies conjugated with nanoparticles (e.g. gold nanoparticles) that are responsible for the visual read-out. The test pad is the location of the read-out. The crucial reaction on the test pad happens within spatially defined zones (i.e. test and control zones) that contain specific recognition elements (i.e. capture and secondary antibody). Single LFDs contain one test zone (i.e. one single test on one strip), while multiplexed LFDs accommodate 2+ test zones (i.e. multiple tests on one single strip). The wick pad acts as bin, prevents backflow of excess reagents, and maintains capillary flow.

Lateral flow devices (LFDs) are composed of four overlapping membranes (sample pad, conjugate release pad, membrane with antibodies (test pad), and adsorbent pad) on a plastic adhesive backing card (Figure 3A). Antibodies that detect specific proteins or molecular markers are imprinted on the membranes. The accuracy of LFAs depends on the specificity and efficacy of antibodies. Multiplexed LFAs (xLFAs) detect multiple markers on the same strip (Figure 3B), enabling more efficient testing, although they are more difficult to design without compromising specificity and sensitivity [72].

LFAs are well established within the agricultural sector for the detection of crop diseases [73]. Although lacking in sensitivity for detecting low-density markers or pathogens in comparison with nucleic acid-based diagnostics tests [74], LFAs are among the fastest and simplest tests available, facilitating rapid in-field decision-making, without the need for equipment.

In addition to disease detection, LFAs can also be used for exploring complex traits that are linked to control. Poor weed control due to herbicide resistance incurs significant costs; for example, herbicide resistant black-grass (*Alopecurus myosuroides*) has been associated with U.K. wheat production losses of 1 million tonnes per year [75]. The quick and reliable diagnosis of herbicide resistance is, therefore, an essential step to improve weed control and minimise adverse effects on crop production and the farm economy [76,77]. For instance, the early detection of a protein biomarker, black-grass glutathione-s-transferase phi (F) 1 and its orthologues in rye-grass (*Lolium rigidum*), barren brome (*Bromus sterilis*), and meadow brome (*Bromus commutatus*) using LFAs confirmed the existent of herbicide resistance in these species [78]. This kind of information is invaluable for growers and consultants to adopt appropriate weed control measures.

There is a growing need for rapid tests such as xLFAs in agriculture for disease and resistance detection, driven by the (re-)emergence of diseases and the need for the more sustainable application of fungicides, insecticides, or herbicides. A digital agronomy system that combines results from LFAs and other tests to deliver an interpretation of results and recommended actions would improve early diagnosis of problems and help with farmer adoption of new technologies.

## Nucleic acid-based detection methods

Detection methods which target a pathogen's nucleic acid are able to target a particular species with very high specificity and have the potential to be extremely sensitive [19,79]. In the laboratory, methods most commonly use polymerase chain reaction (PCR) to amplify a target-specific sequence. Most commonly PCR is implemented in a diagnostic setting as real-time PCR or quantitative PCR (qPCR), to improve throughput and limit post-PCR contamination. These technologies (including the rapid cycling variants [80]) have been in use in plant pests detection for over two decades [81].

PCR-based methods have been integrated into microfluidic ‘lab-on-a-chip’ devices which miniaturise amplification and detection. This approach has been used to develop portable devices for the detection of plant pathogens [82,83]. However, the extraction processes needed to yield nucleic acid free from inhibitors either add complexity to the system or require off-chip processing before testing.

Nucleic acid amplification methods which do not use PCR have potential advantages for field use [84]. Isothermal methods avoid the need for thermal cycling, allowing the use of less complex instruments or even body heat to achieve reaction temperatures. This brings the potential for use in a wider range of environments and integration into automated systems. Loop-mediated isothermal amplification (LAMP), recombinase polymerase amplification (RPA), and helicase dependant amplification (HPA) are isothermal amplification methods which have short reaction times (often 10–30 min) and tolerance of inhibitors co-extracted when simplified sample preparation methods are used, making them well suited to in-field use [85,86].

Nucleic acid-based methods with the potential to be used outside the laboratory have been developed for plant pathogens including viruses, bacteria, fungi, and oomycetes [87]. Most of these methods have been used to test small samples of plant tissue with symptoms of disease with relatively low throughput, to confirm the presence of a specific pest. Crude extraction methods have been developed which are well suited to processing small samples of plant tissue (for example, a single or excised lesion). Expanding the use of on-site nucleic acid testing to applications requiring detection of disease before the observation of symptoms will require the ability to test larger samples, while retaining operational simplicity and a high level of sensitivity.

Simplicity for the end user is greatest in a fully automated system, such as platforms which combines an aerial spore trap with periodic automated DNA-based testing for fungal crop diseases in a standalone instrument.

## DNA sequencing technologies

The ability to sequence all the nucleic acids in a sample allows a non-targeted approach to be taken, asking the question ‘what is present in this sample?' in contrast with targeted assays which ask, ‘is pathogen X present in this sample?'. It is also possible to ask a semi-targeted question such as ‘which bacteria are in this sample?' using metabarcoding, where only specific and taxa-discriminating genes are sequenced. The impact of high-throughput sequencing (HTS) on plant health diagnostics has recently been reviewed [17].

**Figure 4.**
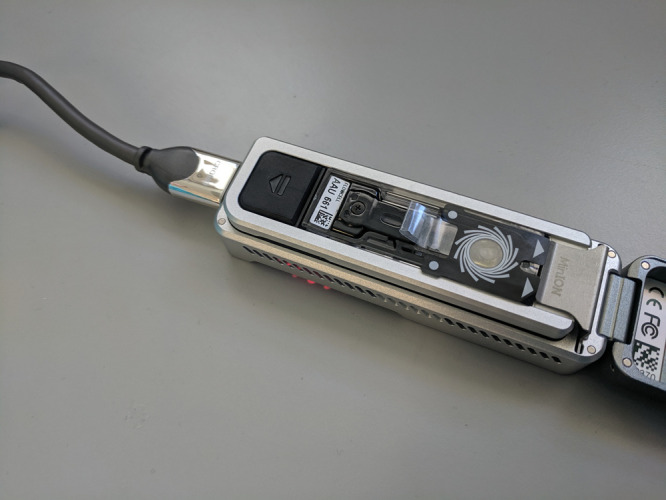
MinION sequencer with Flongle flowcell adapter and mini flowcell fitted. Also shows USB connection which allows the device to be run from a laptop.

HTS platforms produced by companies including Roche, Illumina, and Pacific Biosciences are generally large, complex, and expensive in both capital and reagents costs, and they tend to reside in dedicated sequencing laboratories. In 2014, Oxford Nanopore Technologies (ONT) developed the MinION, the first portable high-throughput sequencer, which can generate up to 30 Gb of data. More information on the MinION can be found at https://nanoporetech.com/. Recent developments involving the Flongle (flowcell adapter) (Figure 4) allow the MinION to use low-cost (< $100) mini flowcells, bringing the potential for use in diagnostic testing in situations where other HTS platforms are impractical or too expensive. The low capital costs of the MinION and the low cost of mini flowcells offers a potential application of this technology in low resource laboratories allowing them to access the genomics revolution without the massive capital outlay of more traditional genome sequencers. Use of the MinION has been demonstrated for the detection of plant-invading bacteria, viruses, fungi, phytoplasma, and insects [88,89] and in virus surveillance in the field in Africa, although the error rate was acknowledged as causing issues with phylogenetic analysis [90].

Most HTS platforms produce short DNA fragments, but the ONT and Pacific Biosciences platforms sequence much larger fragments of DNA, allowing analysis of genotypes present in mixed populations. Radhakrishnan et al. [91] demonstrated that ONT can be used to rapidly identify individual strains of the wheat yellow rust pathogen, *Puccinia striiformis f.sp. tritici* (Pst). Compared with other HTS platforms, ONT technology is uniquely suited for low resource settings, this facilitates an increase in surveillance for strains of this damaging pathogen and enable more precise selection of appropriate varieties. Whilst there are examples of researchers using the ONT platform in the field [90], more work on streamlining the protocols and data analysis is required for it to become a routine field test, used by non-specialists such as farmers or agronomists.

## Potential of synthetic biology for self-reporting

**Figure 5.**
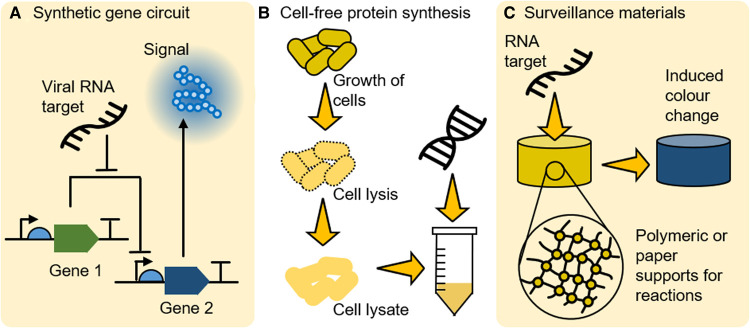
Cell-free synthetic gene circuits. (**A**) Synthetic gene circuits can be designed to produce different outputs in response to a range of biotic and abiotic targets. In this example, the circuit produces a blue signal in response to a viral RNA target. (**B**) Traditionally, such circuitry has been designed and implemented in whole cells. Now, however, it is increasingly common to deploy such circuits in a cell-free manner, by using prepared cell lysates from bacteria to perform these reactions. This obviates the need for the use of genetically modified organisms outside a laboratory. (**C**) Recent developments have also demonstrated that these cell-free reactions are compatible with a range of physical supports, including paper and hydrogel materials, allowing cell-free reactions to be incorporated into these materials and utilise said materials as interaction surfaces. This opens the possibility of developing materials with surveillance capabilities that can have a continuous presence in the field.

Almost all the methods discussed so far, require users to actively interact with the technology. Active monitoring can be laborious and time consuming [92], ultimately limiting uptake. Synthetic biology offers a potential avenue for transitioning from active to passive monitoring via biosensor development [93], where devices are set to monitor the area of interest. Synthetic biology provides a rapid and cost-effective workflow for the development of diagnostic devices through engineering design principals applied to biological systems resulting in short design-build-test cycles [94]. Engineered gene circuits can place complex technology in the hands of nonexpert operators and produce simple, informative outputs that can enable rapid, informed decisions [95] (Figure 5A).

These circuits can be designed following a ‘top down’ (application driven) or ‘bottom up’ (reconstruction) approach [96]. Of relevance to surveillance and in-field diagnostics is a recent shift from traditional cell-based deployment to cell-free systems (Figure 5B). This has the distinct advantage of not requiring the use of genetically modified organisms beyond the laboratory [97].

The use of easy to use, low-cost field diagnostics is already possible. A field-deployable biosensor utilising riboswitches for the detection of fluoride in water samples that produces a visible colour change upon detection of fluoride samples [98] has recently been deployed. The next challenge is to move from diagnostics to surveillance. The potential to take such devices and embed them in materials, such as paper or hydrogels [99], allows such diagnostics to become surveillance systems with a physical presence in the field rather than a complex specialist diagnostic (Figure 5C). The goal is to develop biosensors that can be embedded in the agricultural environment that monitor crop pathogens, or their vectors, and that are robust enough to handle complex samples such as soil or ground water, which can inhibit other diagnostic platforms, and other environmental challenges. In an agricultural context, biosensors could be positioned in fields or storage sites and periodically checked for produced indicators of environmental change, such as pathogen presence.

## Discussion

All the technologies discussed have the potential to enhance the detection of pests and demonstrate a great potential to support plant biosecurity and surveillance. However, no single technology can be used for all pests in all scenarios. The adoption of an interdisciplinary approach from the outset is important to achieve overall aims. Bringing together scientists from different disciplines along with stakeholders (inspectors, extension officers, industry, and farmers) to facilitate efficient cocreation will ensure that technologies being designed are fit-for-purpose and should facilitate rapid deployment trajectories that are paramount for success.

A significant benefit of the technologies described in this review is that they all provide an opportunity to move diagnostics away from centralised reference laboratories, providing data directly to end users either in the field or in remote or less well-equipped laboratories. As a result, it is crucial that field inspectors, farmers and other users of the technology are prepared to deal with the results and make rapid decisions effectively. Currently, there is a gap for some technologies where the data outputs are complex and not easily interpreted by end users. Other technologies such as the PlantVillage Nuru app include interpretation of data through to the point of recommending actions. The use of smartphones as an approach to turn data into actionable knowledge is a trend likely to accelerate, helping with the adoption of new technologies by end user communities.

**Figure 6.**
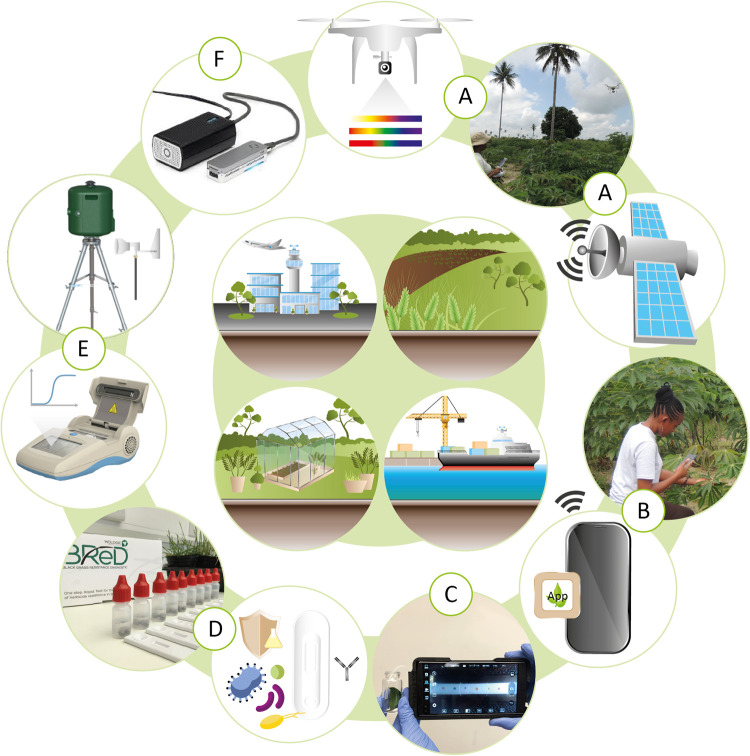
Integration of different technologies to support plant biosecurity and surveillance. Centre: two main scenarios are represented; biosecurity (port and airport) and in-field surveillance (glasshouse and field). The technologies represented are (**A**) remote sensing; (**B**) smartphone-based detection; (**C**) plant VOCs detection; (**D**) LFA detection of proteins, (**E**) targeted and automated DNA detection and (**F**) High-throughput sequencing technologies.

Deploying multiple technologies to create an overall surveillance system is also likely to be an important future trend (Figure 6), since no single technology can provide all the solutions to achieve effective disease surveillance outcomes. As an example, satellite or drone imaging data could be used to locate areas of crops which are experiencing biotic and/or abiotic stress. Although the data may not be sufficiently specific to identify individual pests, it can be used to direct the attention of growers or inspectors to the stressed plants for further analysis. In the field, smartphone technology could be used to screen large numbers of samples and provide a diagnosis based on symptoms. In situations where species or strain level information is required, the smartphone app could identify the best samples to test using high specificity LFA or DNA technologies, reducing the number of samples to be analysed and potentially providing a ‘triage’ of which additional tests should be used. This strategy would accelerate the decision-making process and timely deployment of mitigation measures.

Integration of technologies and combining data from different platforms to achieve an overall aim, rather than using them sequentially or in tandem, may also be desirable. Furthermore, combining data on pests (presence, abundance, strain, resistance genotype, etc.), with information on actions taken by end users (for example, the spray programme used) and subsequent outcomes (i.e. effectiveness of a management action) could facilitate the use of machine learning algorithms to modify and improve on the recommended action for the control of specific pests, ultimately creating a ‘digital agronomist’ that can act on data and is able to learn from the outcomes of those actions. Combining data to facilitate complex decision-making, however, may need remote data analysis due to the computing power required. The lack of internet access in rural communities may hinder the uptake of these approaches, and this should be addressed to maximise accessibility.

Automation will be crucial in building effective surveillance systems to provide early warning of the presence of a pest. End users such as farmers, inspectors and extension officers are typically time poor, and expecting them to invest large amounts of time into surveillance for pests is unrealistic except for the highest value problems. This is likely to reduce uptake and drive the adoption of smaller surveillance networks, undermining the value of the results from the system. The use of automation and wireless data transfer should improve the uptake of the technologies, though it will also likely create a trade-off, where reduced time input necessitates greater capital expenditure associated with automated systems.

A distinction can be made between methods regarding capital and running costs. Some methods require the purchase of capital equipment (e.g. VOC, image, and spectral analysis platforms) but have no or limited requirement for consumables. Thus the upfront cost facilitates the testing of many more samples. Other techniques (e.g. LFAs) have no requirement for capital equipment, and every test generates a cost to the user. Finally, there are approaches that have both capital and on-going consumable costs (e.g. DNA methods such as HTS or LAMP), where the manufacturer can build a pricing structure. The co-creation process should seek to determine whether capital and/or running costs could form an insurmountable barrier to adoption for the intended end users.

Methods that require many tests to be performed are often used to provide a time series or spatial representation of the presence/absence of a pest, and much of the cost necessarily derives from tests which yield negative results. Sensors that are triggered only upon the presence of the pathogen would be hugely beneficial for these scenarios. This is something that may be achievable using synthetic biology approaches, mimicking the early processes that enable a plant to recognize pests. Harnessing these interactions in a self-reporting system would create a sensor that reports only in the presence of the pest, without on-going consumables costs while the pest is absent.

Innovations in technology are providing a great array of tools to enable surveillance in support of biosecurity and control of endemic pests. These have been driven forward by various advances including the availability of portable platforms for DNA analysis, accessibility of remote spectral data at a meaningful resolution and the availability of smartphones for analysing and disseminating information to end users. Future developments will be driven by the need for automated/real-time data collection and improved interpretation (i.e. actions not risks). Adoption will depend on improved engagement with end users and economic data on the benefits of increased use of the knowledge provided by surveillance systems in farming and biosecurity.

## Summary

- Plant pests and pathogens cause significant economic losses and pose a significant constraint to agricultural productivity.
- Innovations in technology are providing a great array of tools to enable surveillance in support of biosecurity and control of endemic pests.
- Integration of technologies and combining data from different platforms is paramount to create an overall surveillance system.
- Automation will be crucial in building effective surveillance systems to provide early warning of the presence of a pest.

## Abbreviations

HTS: high-throughput sequencing
LAMP: loop-mediated isothermal amplification
LFAs: lateral flow assays
NDVI: normalised difference vegetation index
ONT: Oxford Nanopore Technologies
SWIR: shortwave infrared
VOC: volatile organic compounds

## References

[1] United Nations. World Population Prospects: The 2015 Revision. 2015

[2] Cassidy, E.S., West, P.C., Gerber, J.S. and Foley, J.A. (2013) Redefining agricultural yields: from tonnes to people nourished per hectare. Environ. Res. Lett. 8, 034015 10.1088/1748-9326/8/3/034015

[3] Mumford, R.A., Macarthur, R. and Boonham, N. (2016) The role and challenges of new diagnostic technology in plant biosecurity. Food Secur. 8, 103–109 10.1007/s12571-015-0533-y

[4] Bebber, D.P. (2015) Range-expanding pests and pathogens in a warming world. Annu. Rev. Phytopathol. 53, 335–356 10.1146/annurev-phyto-080614-120207 26047565

[5] Fox, A., Fowkes, A.R., Skelton, A., Harju, V., Buxton-Kirk, A., Kelly, M. et al. (2019) Using high-throughput sequencing in support of a plant health outbreak reveals novel viruses in *Ullucus tuberosus* (Basellaceae). Plant Pathol. 68, 576–587 10.1111/ppa.12962

[6] Bebber, D.P., Field, E., Gui, H., Mortimer, P., Holmes, T. and Gurr, S.J. (2019) Many unreported crop pests and pathogens are probably already present. Glob. Chang Biol. 25, 2703–2713 10.1111/gcb.14698 31237022

[7] Jones, R.A.C. and Naidu, R.A. (2019) Global dimensions of plant virus diseases: current status and future perspectives. Annu. Rev. Virol. 6, 387–409 10.1146/annurev-virology-092818-015606 31283443

[8] Rubio, L., Galipienso, L. and Ferriol, I. (2020) Detection of plant viruses and disease management: relevance of genetic diversity and evolution. Front. Plant Sci. 11, 1–23 10.3389/fpls.2020.01092 32765569 PMC7380168

[9] Spence, N., Hill, L. and Morris, J. (2020) How the global threat of pests and diseases impacts plants, people, and the planet. PLANTS, PEOPLE, PLANET. Plants People Planet 2, 5–13 10.1002/ppp3.10088

[10] Day, R., Abrahams, P., Bateman, M., Beale, T., Clottey, V., Cock, M. et al. (2017) Fall armyworm: impacts and implications for Africa. Outlooks Pest Manag. 28, 196–201 10.1564/v28_oct_02

[11] Saponari, M., Boscia, D., Nigro, F. and Martelli, G.P. (2013) Identification of DNA sequences related to *Xylella fastidiosa* in oleander, almond and olive trees exhibiting leaf scorch symptoms in Apulia (Southern Italy). J. Plant Pathol. 95, 668 10.4454/JPP.V95I3.035

[12] Almeida, R.P.P. (2018) Emerging plant disease epidemics: biological research is key but not enough. PLoS Biol. 16, e2007020 10.1371/journal.pbio.2007020 30133434 PMC6122826

[13] Kyrkou, I., Pusa, T., Ellegaard-Jensen, L., Sagot, M.F. and Hansen, L.H. (2018) Pierce's disease of grapevines: a review of control strategies and an outline of an epidemiological model. Front. Microbiol. 9, 1–23 10.3389/fmicb.2018.02141 30258423 PMC6143690

[14] Haverkort, A.J., Boonekamp, P.M., Hutten, R., Jacobsen, E., Lotz, L.A.P., Kessel, G.J.T. et al. (2008) Societal costs of late blight in potato and prospects of durable resistance through cisgenic modification. Potato Res. 51, 47–57 10.1007/s11540-008-9089-y

[15] Boonham, N., Kreuze, J., Winter, S., van der Vlugt, R., Bergervoet, J., Tomlinson, J. et al. (2014) Methods in virus diagnostics: from ELISA to next generation sequencing. Virus Res. 186, 20–31 10.1016/j.virusres.2013.12.007 24361981

[16] Adams, I.P., Miano, D.W., Kinyua, Z.M., Wangai, A., Kimani, E., Phiri, N. et al. (2013) Use of next-generation sequencing for the identification and characterization of *Maize chlorotic mottle virus* and *Sugarcane mosaic virus* causing maize lethal necrosis in Kenya. Plant Pathol. 62, 741–749 10.1111/j.1365-3059.2012.02690.x

[17] Adams, I.P., Fox, A., Boonham, N., Massart, S. and De Jonghe, K. (2018) The impact of high throughput sequencing on plant health diagnostics. Eur. J. Plant Pathol. 152, 909–919 10.1007/s10658-018-1570-0

[18] Massart, S., Candresse, T., Gil, J., Lacomme, C., Predajna, L., Ravnikar, M. et al. (2017) A framework for the evaluation of biosecurity, commercial, regulatory, and scientific impacts of plant viruses and viroids identified by NGS technologies. Front. Microbiol. 8, 45 10.3389/fmicb.2017.00045 28174561 PMC5258733

[19] Mumford, R., Boonham, N., Tomlinson, J. and Barker, I. (2006) Advances in molecular phytodiagnostics - new solutions for old problems. Eur. J. Plant Pathol. 116, 1–19 10.1007/s10658-006-9037-0 32214677 PMC7087944

[20] Donoso, A. and Valenzuela, S. (2018) In-field molecular diagnosis of plant pathogens: recent trends and future perspectives. Plant Pathol. 67, 1451–1461 10.1111/ppa.12859

[21] Nilsson, H.E. (1995) Remote sensing and image analysis in plant pathology. Can. J. Plant Pathol. 17, 154–166 10.1080/07060669509500707 18999971

[22] Mahlein, A.K., Kuska, M.T., Behmann, J., Polder, G. and Walter, A. (2018) Hyperspectral sensors and imaging technologies in phytopathology: state of the art. Annu. Rev. Phytopathol. 56, 535–558 10.1146/annurev-phyto-080417-050100 30149790

[23] Nagarajan, S. (1984) Monitoring wheat rust epidemics with the Landsat-2 satellite. Phytopathology 74, 585 10.1094/phyto-74-585

[24] Jackson, R.D. (1986) Remote sensing of biotic and abiotic plant stress. Annu. Rev. Phytopathol. 24, 265–287 10.1146/annurev.py.24.090186.001405

[25] Hatfield, P.L. and Pinter, P.J. (1993) Remote sensing for crop protection. Crop Prot. 12, 403–413 10.1016/0261-2194(93)90001-Y

[26] Yuan, L., Pu, R., Zhang, J., Wang, J. and Yang, H. (2016) Using high spatial resolution satellite imagery for mapping powdery mildew at a regional scale. Precis. Agric. 17, 332–348 10.1007/s11119-015-9421-x

[27] Raza, M.M., Harding, C., Liebman, M. and Leandro, L.F. (2020) Exploring the potential of high-resolution satellite imagery for the detection of soybean sudden death syndrome. Remote Sens. 12, 1213 10.3390/rs12071213

[28] MacDonald, S.L., Staid, M., Staid, M. and Cooper, M.L. (2016) Remote hyperspectral imaging of grapevine leafroll-associated virus 3 in cabernet sauvignon vineyards. Comput. Electron. Agric. 130, 109–117 10.1016/j.compag.2016.10.003

[29] Heim, R.H.J., Wright, I.J., Scarth, P., Carnegie, A.J., Taylor, D. and Oldeland, J. (2019) Multispectral, aerial disease detection for myrtle rust (*Austropuccinia psidii*) on a lemon myrtle plantation. Drones 3, 25 10.3390/drones3010025

[30] Barbedo, J.G.A. (2019) A review on the use of unmanned aerial vehicles and imaging sensors for monitoring and assessing plant stresses. Drones 3, 40 10.3390/drones3020040

[31] Gongora-Canul, C., Salgado, J., Singh, D., Cruz, A., Cotrozzi, L., Couture, J.J. et al. (2019) Temporal dynamics of wheat blast epidemics and agreement between remotely sensed data measurements and visual estimations of wheat spike blast (WSB) under field conditions. Phytopathology 110, 393–405 10.1094/PHYTO-08-19-0297-R 31532351

[32] Hornero, A., Hernández-Clemente, R., North, P.R.J., Beck, P.S.A., Boscia, D., Navas-Cortes, J.A. et al. (2020) Monitoring the incidence of *Xylella fastidiosa* infection in olive orchards using ground-based evaluations, airborne imaging spectroscopy and Sentinel-2 time series through 3-D radiative transfer modelling. Remote Sens. Environ. 236, 111480 10.1016/j.rse.2019.111480

[33] Zarco-Tejada, P.J., Camino, C., Beck, P.S.A., Calderon, R., Hornero, A., Hernández-Clemente, R. et al. (2018) Previsual symptoms of *Xylella fastidiosa* infection revealed in spectral plant-trait alterations. Nat. Plants 4, 432–439 10.1038/s41477-018-0189-7 29942047

[34] Fallon, B., Yang, A., Lapadat, C., Armour, I., Juzwik, J., Montgomery, R.A. et al. (2020) Spectral differentiation of oak wilt from foliar fungal disease and drought is correlated with physiological changes. Tree Physiol. 40, 377–390 10.1093/treephys/tpaa005 32031662

[35] Gold, K.M., Townsend, P.A., Larson, E.R., Herrmann, I. and Gevens, A.J. (2020) Contact reflectance spectroscopy for rapid, accurate, and nondestructive *Phytophthora infestans* clonal lineage discrimination. Phytopathology 110, 851–862 10.1094/PHYTO-08-19-0294-R 31880984

[36] Gold, K.M., Townsend, P.A., Herrmann, I. and Gevens, A.J. (2020) Investigating potato late blight physiological differences across potato cultivars with spectroscopy and machine learning. Plant Sci. 295, 110316 10.1016/j.plantsci.2019.110316 32534618

[37] Bienkowski, D., Aitkenhead, M.J., Lees, A.K., Gallagher, C. and Neilson, R. (2019) Detection and differentiation between potato (*Solanum tuberosum*) diseases using calibration models trained with non-imaging spectrometry data. Comput. Electron. Agric. 167, 105056 10.1016/j.compag.2019.105056

[38] Abdulridha, J., Ehsani, R., Abd-Elrahman, A. and Ampatzidis, Y. (2019) A remote sensing technique for detecting laurel wilt disease in avocado in presence of other biotic and abiotic stresses. Comput. Electron. Agric. 156, 549–557 10.1016/j.compag.2018.12.018

[39] Gold, K.M., Townsend, P.A., Chlus, A., Herrmann, I., Couture, J.J., Larson, E.R. et al. (2020) Hyperspectral measurements enable pre-symptomatic detection and differentiation of contrasting physiological effects of late blight and early blight in potato. Remote Sens. 12, 286 10.3390/rs12020286

[40] Barbedo JG, A. (2013) Digital image processing techniques for detecting, quantifying and classifying plant diseases. Springerplus 2, 660 10.1186/2193-1801-2-660 24349961 PMC3863396

[41] Lowe, A., Harrison, N. and French, A.P. (2017) Hyperspectral image analysis techniques for the detection and classification of the early onset of plant disease and stress. Plant Methods 13, 80 10.1186/s13007-017-0233-z 29051772 PMC5634902

[42] Petrellis, N. (2018) A review of image processing techniques common in human and plant disease diagnosis. Symmetry (Basel) 10, 270 10.3390/sym10070270

[43] Pethybridge, S.J. and Nelson, S.C. (2015) Leaf doctor: a new portable application for quantifying plant disease severity. Plant Dis. 99, 1310–1316 10.1094/PDIS-03-15-0319-RE 30690990

[44] Ramcharan, A., Baranowski, K., McCloskey, P., Ahmed, B., Legg, J. and Hughes, D.P. (2017) Deep learning for image-based cassava disease detection. Front. Plant Sci. 8, 1852 10.3389/fpls.2017.01852 29163582 PMC5663696

[45] Ramcharan, A., McCloskey, P., Baranowski, K., Mbilinyi, N., Mrisho, L., Ndalahwa, M. et al. (2019) A mobile-based deep learning model for cassava disease diagnosis. Front. Plant Sci. 10, 272 10.3389/fpls.2019.00272 30949185 PMC6436463

[46] Selvaraj, M.G., Vergara, A., Ruiz, H., Safari, N., Elayabalan, S., Ocimati, W. et al. (2019) AI-powered banana diseases and pest detection. Plant Methods 15, 92 10.1186/s13007-019-0475-z

[47] Mrisho, L.M., Mbilinyi, N.A., Ndalahwa, M., Ramcharan, A.M., Kehs, A.K., McCloskey, P.C. et al. (2020) Accuracy of a smartphone-based object detection model, PlantVillage Nuru, in identifying the foliar symptoms of the viral diseases of cassava–CMD and CBSD. Front. Plant Sci. 11, 590889 10.3389/fpls.2020.590889 33391304 PMC7775399

[48] Aksenov, A.A., Novillo, A.V.G., Sankaran, S., Fung, A.G., Pasamontes, A., Martinelli, F. et al. (2013) Volatile organic compounds (VOCs) for noninvasive plant diagnostics. ACS Symp. Ser. 1141, 73–95 10.1021/bk-2013-1141.ch006

[49] Jansen, R.M.C., Wildt, J., Hofstee, J.W., Bouwmeester, H.J. and van Henten, E.J. (2010) Plant volatiles: useful signals to monitor crop health status in greenhouses. Plant Commun. Ecol. Perspect. 229–247 10.1007/978-3-642-12162-3_13

[50] Jansen, R.M.C., Wildt, J., Kappers, I.F., Bouwmeester, H.J., Hofstee, J.W. and Van Henten, E.J. (2011) Detection of diseased plants by analysis of volatile organic compound emission. Annu. Rev. Phytopathol. 49, 157–174 10.1146/annurev-phyto-072910-095227 21663436

[51] Jansen, R.M.C., Hofstee, J.W., Wildt, J., Verstappen, F.W.A., Bouwmeester, H.J. and van Henten, E.J. (2009) Induced plant volatiles allow sensitive monitoring of plant health status in greenhouses. Plant Signal. Behav. 4, 824–829 10.4161/psb.4.9.9431 19847108 PMC2802792

[52] Shulaev, V., Silverman, P. and Raskin, I. (1997) Airborne signalling by methyl salicylate in plant pathogen resistance. Nature 385, 718–721 10.1038/385718a0

[53] Tholl, D., Boland, W., Hansel, A., Loreto, F., Röse, U.S.R. and Schnitzler, J.P. (2006) Practical approaches to plant volatile analysis. Plant J. 45, 540–560 10.1111/j.1365-313X.2005.02612.x 16441348

[54] Miller, R.A., Nazarov, E.G. Eiceman, G.A. and King, A.T. (2001) A MEMS radio-frequency ion mobility spectrometer for chemical vapor detection. Sens. Actuators A Phys. 91, 301–312 10.1016/S0924-4247(01)00600-8

[55] Contreras, J.A., Murray, J.A., Tolley, S.E., Oliphant, J.L., Tolley, H.D., Lammert, S.A. et al. (2008) Hand-portable gas chromatograph-toroidal ion trap mass spectrometer (GC-TMS) for detection of hazardous compounds. J. Am. Soc. Mass Spectrom. 19, 1425–1434 10.1016/j.jasms.2008.06.022 18672381

[56] Anishchenko, I.M., McCartney, M.M., Fung, A.G., Peirano, D.J., Schirle, M.J., Kenyon, N.J. et al. (2018) Modular and reconfigurable gas chromatography/differential mobility spectrometry (GC/DMS) package for detection of volatile organic compounds (VOCs). Int. J. Ion Mobil. Spectrom. 21, 125–136 10.1007/s12127-018-0240-4 31086501 PMC6510507

[57] Sharma, R., Zhou, M., Hunter, M.D. and Fan, X. (2019) Rapid in situ analysis of plant emission for disease diagnosis using a portable Gas chromatography device. J. Agric. Food Chem. 67, 7530–7537 10.1021/acs.jafc.9b02500 31184878

[58] Arshak, K., Moore, E., Lyons, G.M., Harris, J. and Clifford, S. (2004) A review of gas sensors employed in electronic nose applications. Sens Rev. 24, 181–198 10.1108/02602280410525977

[59] Nagle, H.T., Schiffman, S.S. and Gutierrez-Osuna, R. (1998) How and why of electronic noses. IEEE Spectr. 35, 22–31 10.1109/6.715180

[60] Wilson, A.D. and Baietto, M. (2009) Applications and advances in electronic-nose technologies. Sensors 9, 5099–5148 10.3390/s90705099 22346690 PMC3274163

[61] Wilson, A.D., Lester, D.G. and Oberle, C.S. (2004) Development of conductive polymer analysis for the rapid detection and identification of phytopathogenic microbes. Phytopathology 94, 419–431 10.1094/PHYTO.2004.94.5.419 18943759

[62] Spinelli, F., Noferini, M., Vanneste, J.L. and Costa, G. (2010) Potential of the electronic-nose for the diagnosis of bacterial and fungal diseases in fruit trees. EPPO Bull. 40, 59–67 10.1111/j.1365-2338.2009.02355.x

[63] Cellini, A., Biondi, E., Blasioli, S., Rocchi, L., Farneti, B., Braschi, I. et al. (2016) Early detection of bacterial diseases in apple plants by analysis of volatile organic compounds profiles and use of electronic nose. Ann. Appl. Biol. 168, 409–420 10.1111/aab.12272

[64] Cheng, S.-M., Wang, J., Wang, Y.-W. and Wei, Z.-B. (2017) Discrimination of different types damage of tomato seedling by electronic nose. Chin. J. Sens. Actuators 25:1184–1188 10.1051/itmconf/20171101019

[65] Li, Z., Paul, R., Ba Tis, T., Saville, A.C., Hansel, J.C., Yu, T. et al. (2019) Non-invasive plant disease diagnostics enabled by smartphone-based fingerprinting of leaf volatiles. Nat. Plants 5, 856–866 10.1038/s41477-019-0476-y 31358961

[66] Li, Z., Askim, J.R. and Suslick, K.S. (2019) The optoelectronic nose: colorimetric and fluorometric sensor arrays. Chem. Rev. 119, 231–292 10.1021/acs.chemrev.8b00226 30207700

[67] Askim, J.R., Mahmoudi, M. and Suslick, K.S. (2013) Optical sensor arrays for chemical sensing: The optoelectronic nose. Chem. Soc. Rev. 42, 8649 10.1039/c3cs60179j 24091381

[68] Im, H., Lee, S., Naqi, M., Lee, C. and Kim, S. (2018) Flexible PI-based plant drought stress sensor for real-time monitoring system in smart farm. Electron 7, 114 10.3390/electronics7070114

[69] Lan, L., Le, X., Dong, H., Xie, J., Ying, Y. and Ping, J. (2020) One-step and large-scale fabrication of flexible and wearable humidity sensor based on laser-induced graphene for real-time tracking of plant transpiration at bio-interface. Biosens. Bioelectron. 165, 112360 10.1016/j.bios.2020.112360 32729493

[70] Lee, K., Park, J., Lee, M.S., Kim, J., Hyun, B.G., Kang, D.J. et al. (2014) In-situ synthesis of carbon nanotube-graphite electronic devices and their integrations onto surfaces of live plants and insects. Nano Lett. 14, 2647–2654 10.1021/nl500513n 24742260

[71] Koczula, K.M. and Gallotta, A. (2016) Lateral flow assays. Essays Biochem. 60, 111–120 10.1042/EBC20150012 27365041 PMC4986465

[72] Hanafiah K, M., Arifin, N., Bustami, Y., Noordin, R., Garcia, M. and Anderson, D. (2017) Development of multiplexed infectious disease lateral flow assays: challenges and opportunities. Diagnostics 7, 51 10.3390/diagnostics7030051 PMC5617951 28880218

[73] Grothaus, G.D., Bandla, M., Currier, T., Giroux, R., Jenkins, G.R., Lipp, M. et al. (2006) Immunoassay as an analytical tool in agricultural biotechnology. J. AOAC Int. 89, 913–992 10.1093/jaoac/89.4.913 16915826

[74] Lalremruata, A., Nguyen, T.T., McCall, M.B.B., Mombo-Ngoma, G., Agnandji, S.T., Adegnika, A.A. et al. (2020) Recombinase polymerase amplification and lateral flow assay for ultrasensitive detection of low-density *Plasmodium falciparum* infection from controlled human malaria infection studies and naturally acquired infections. J. Clin. Microbiol. 58, e01879-19 10.1128/JCM.01879-19 32102854 PMC7180247

[75] Varah, A., Ahodo, K., Coutts, S.R., Hicks, H.L., Comont, D., Crook, L. et al. (2020) The costs of human-induced evolution in an agricultural system. Nat. Sustain. 3, 63–71 10.1038/s41893-019-0450-8 31942455 PMC6962049

[76] Davies, L.R., Onkokesung, N., Brazier-Hicks, M., Edwards, R. and Moss, S. (2020) Detection and characterization of resistance to acetolactate synthase inhibiting herbicides in *Anisantha* and *Bromus* species in the United Kingdom. Pest Manag. Sci. 76, 2473–2482 10.1002/ps.5788 32061023

[77] Comont, D., Lowe, C., Hull, R., Crook, L., Hicks, H.L., Onkokesung, N. et al. (2020) Evolution of generalist resistance to herbicide mixtures reveals a trade-off in resistance management. Nat. Commun. 11, 3086 10.1038/s41467-020-16896-0 32555156 PMC7303185

[78] Edwards, R. and Onkokesung, N. (2020) Resisting resistance: new applications for molecular diagnostics in crop protection. Biochem (Lond) 42, 6–12 10.1042/BIO20200040

[79] Boonham, N., Glover, R., Tomlinson, J. and Mumford, R. (2008) Exploiting generic platform technologies for the detection and identification of plant pathogens. Eur. J. Plant Pathol. 121, 355–363 10.1007/s10658-008-9284-3

[80] Tomlinson, J.A., Boonham, N., Hughes, K.J.D., Griffin, R.L. and Barker, I. (2005) On-site DNA extraction and real-time PCR for detection of *Phytophthora ramorum* in the field. Appl. Environ. Microbiol. 71, 6702–6710 10.1128/AEM.71.11.6702-6710.2005 16269700 PMC1287659

[81] Weller, S.A., Elphinstone, J.G., Smith, N.C., Boonham, N. and Stead, D.E. (2000) Detection of *Ralstonia solanacearum* strains with a quantitative, multiplex, real-time, fluorogenic PCR (TaqMan) assay. Appl. Environ. Microbiol. 66, 2853–2858 10.1128/AEM.66.7.2853-2858.2000 10877778 PMC92083

[82] Julich, S., Riedel, M., Kielpinski, M., Urban, M., Kretschmer, R., Wagner, S. et al. (2011) Development of a lab-on-a-chip device for diagnosis of plant pathogens. Biosens. Bioelectron. 26, 4070–4075 10.1016/j.bios.2011.03.035 21531125

[83] Koo, C., Malapi-Wight, M., Kim, H.S., Cifci, O.S., Vaughn-Diaz, V.L., Ma, B. et al. (2013) Development of a real-time microchip PCR system for portable plant disease diagnosis. PLoS One 8, e82704 10.1371/journal.pone.0082704 24349341 PMC3861469

[84] Lau, H.Y. and Botella, J.R. (2017) Advanced DNA-based point-of-care diagnostic methods for plant diseases detection. Front. Plant Sci. 8, 2016 10.3389/fpls.2017.02016 29375588 PMC5770625

[85] Silva, G., Oyekanmi, J., Nkere, C.K., Bömer, M., Kumar, P.L. and Seal, S.E. (2018) Rapid detection of potyviruses from crude plant extracts. Anal. Biochem. 546, 17–22 10.1016/j.ab.2018.01.019 29378167 PMC5873530

[86] Nkere, C.K., Oyekanmi, J.O., Silva, G., Bömer, M., Atiri, G.I., Onyeka, J. et al. (2018) Chromogenic detection of yam mosaic virus by closed-tube reverse transcription loop-mediated isothermal amplification (CT-RT-LAMP). Arch. Virol. 163, 1057–1061 10.1007/s00705-018-3706-0 29308543 PMC5854734

[87] Paul, R., Ostermann, E. and Wei, Q. (2020) Advances in point-of-care nucleic acid extraction technologies for rapid diagnosis of human and plant diseases. Biosens. Bioelectron. 169, 112592 10.1016/j.bios.2020.112592 32942143 PMC7476893

[88] Chalupowicz, L., Dombrovsky, A., Gaba, V., Luria, N., Reuven, M., Beerman, A. et al. (2019) Diagnosis of plant diseases using the nanopore sequencing platform. Plant Pathol. 68, 229–238 10.1111/ppa.12957

[89] Badial, A.B., Sherman, D., Stone, A., Gopakumar, A., Wilson, V., Schneider, W. et al. (2018) Nanopore sequencing as a surveillance tool for plant pathogens in plant and insect tissues. Plant Dis. 102, 1648–1652 10.1094/PDIS-04-17-0488-RE 30673417

[90] Boykin, L.M., Sseruwagi, P., Alicai, T., Ateka, E., Mohammed, I.U., Stanton, J.A.L. et al. (2019) Tree lab: Portable genomics for early detection of plant viruses and pests in sub-saharan africa. Genes (Basel) 10, 632 10.3390/genes10090632 PMC6769854 31438604

[91] Radhakrishnan G, V., Cook, N.M., Bueno-Sancho, V., Lewis, C.M., Persoons, A., Mitiku, A.D. et al. (2019) MARPLE, a point-of-care, strain-level disease diagnostics and surveillance tool for complex fungal pathogens. BMC Biol. 17, 65 10.1186/s12915-019-0684-y 31405370 PMC6691556

[92] Slomovic, S., Pardee, K. and Collins, J.J. (2015) Synthetic biology devices for in vitro and in vivo diagnostics. Proc. Natl Acad. Sci. U.S.A. 112, 14429–14435 10.1073/pnas.1508521112 26598662 PMC4664311

[93] Tinafar, A., Jaenes, K. and Pardee, K. (2019) Synthetic biology goes cell-free. BMC Biol. 17, 64 10.1186/s12915-019-0685-x 31395057 PMC6688370

[94] Oberortner, E., Evans, R., Meng, X., Nath, S., Plahar, H., Simirenko, L. et al. (2020) An integrated computer-aided design and manufacturing workflow for synthetic biology. Methods Mol. Biol. 2205, 3–18 10.1007/978-1-0716-0908-8_1 32809190

[95] Courbet, A., Renard, E. and Molina, F. (2016) Bringing next-generation diagnostics to the clinic through synthetic biology. EMBO Mol. Med. 8, 987–991 10.15252/emmm.201606541 27402339 PMC5009805

[96] Ausländer, S., Ausländer, D. and Fussenegger, M. (2017) Synthetic biology—the synthesis of biology. Angew Chem. – Int. Ed. 56, 6396–6419 10.1002/anie.201609229 27943572

[97] Karig, D.K. (2017) Cell-free synthetic biology for environmental sensing and remediation. Curr. Opin. Biotechnol. 45, 69–75 10.1016/j.copbio.2017.01.010 28226291

[98] Thavarajah, W., Silverman, A.D., Verosloff, M.S., Kelley-Loughnane, N., Jewett, M.C. and Lucks, J.B. (2020) Point-of-use detection of environmental fluoride *via* a cell-free riboswitch-based biosensor. ACS Synth .Biol. 9, 10–18 10.1021/acssynbio.9b00347 31829623 PMC7412506

[99] Whitfield, C.J., Banks, A.M., Dura, G., Love, J., Fieldsend, J.E., Goodchild, S.A. et al. (2020) Cell-free protein synthesis in hydrogel materials. Chem. Commun. 56, 7108–7111 10.1039/d0cc02582h 32458833

